# Over‐the‐top plus lateral extra‐articular tenodesis anterior cruciate ligament reconstruction: Ten tips for success

**DOI:** 10.1002/jeo2.70785

**Published:** 2026-05-25

**Authors:** Stefano Zaffagnini, Ying Ren Mok, Alberto Grassi, Giulio Maria Marcheggiani Muccioli

**Affiliations:** ^1^ Clinica Ortopedica e Traumatologica II, IRCCS Istituto Ortopedico Rizzoli Bologna Italy; ^2^ Dipartimento di Scienze Biomediche e Neuromotorie (DIBINEM) University of Bologna Bologna Italy; ^3^ Department of Orthopaedic Surgery National University Hospital Singapore Singapore

**Keywords:** ACL reconstruction, hamstrings, lateral extra‐articular tenodesis, over‐the‐top, technique

## Abstract

**Purpose:**

Over‐the‐top (OTT) plus Lateral Extra‐articular Tenodesis (LET) anterior cruciate ligament (ACL) reconstruction has been performed continuously at the Rizzoli Orthopedic Institute since 1993, with approximately 6000 procedures completed and long‐term follow‐up reported up to 25 years. Despite its durability and reproducibility, detailed technical updates have been limited. The purpose of this review is to summarise 10 practical tips aimed at preventing and managing intra‐operative complications during OTT ACL reconstruction.

**Methods:**

This technical review is based on three decades of cumulative institutional experience with OTT plus LET ACL reconstruction across diverse patient populations, including skeletally immature patients, amateur athletes, elite professional soccer players and revision cases. Key technical steps are presented in a structured ‘Ten Tips’ format, emphasising strategies to preserve graft biology, ensure anatomic positioning and avoid intra‐operative pitfalls.

**Results:**

Critical elements include preservation of distal hamstring vascularity, anatomic tibial tunnel placement, meticulous preparation of the OTT femoral pathway, safe lateral window development with protection of posterior structures, controlled graft passage to avoid clumping or kinking, precise anterior‐distal staple insertion to prevent posterior femoral cortical fracture and standardised completion of the LET. Attention to these principles minimises technical errors, protects the neurovascular bundle and PCL and promotes optimal graft orientation as demonstrated by magnetic resonance imaging‐based inclination parameters.

**Conclusion:**

When performed with meticulous technique and adherence to reproducible surgical principles, OTT plus LET ACL reconstruction remains a versatile, economical and biologically favourable procedure capable of achieving anatomic graft positioning while avoiding femoral tunnel–related complications. These 10 technical recommendations provide a structured framework for reducing intra‐operative complications and optimising outcomes.

**Level of Evidence:**

Level V, expert opinion.

**Abbreviations**
ACL: anterior cruciate ligament
OTT: over‐the‐top

## INTRODUCTION

Over‐the‐top (OTT) anterior cruciate ligament (ACL) reconstruction with Lateral Extra‐articular Tenodesis (LET) has been performed at the Rizzoli Orthopedic Institute since 1993, with the original technique formally described in 1998 by Marcacci and Zaffagnini [16] and remaining largely unchanged for over three decades. During this period, approximately 6000 procedures have been performed, with excellent outcomes reported at 5‐ [17], 10‐ [7, 15], 20‐ [29] and 25‐year [28] follow‐up in diverse populations, including adolescents [8] and elite professional soccer players [1]. These results demonstrate that the technique has stood the test of time.

**Figure 1:**
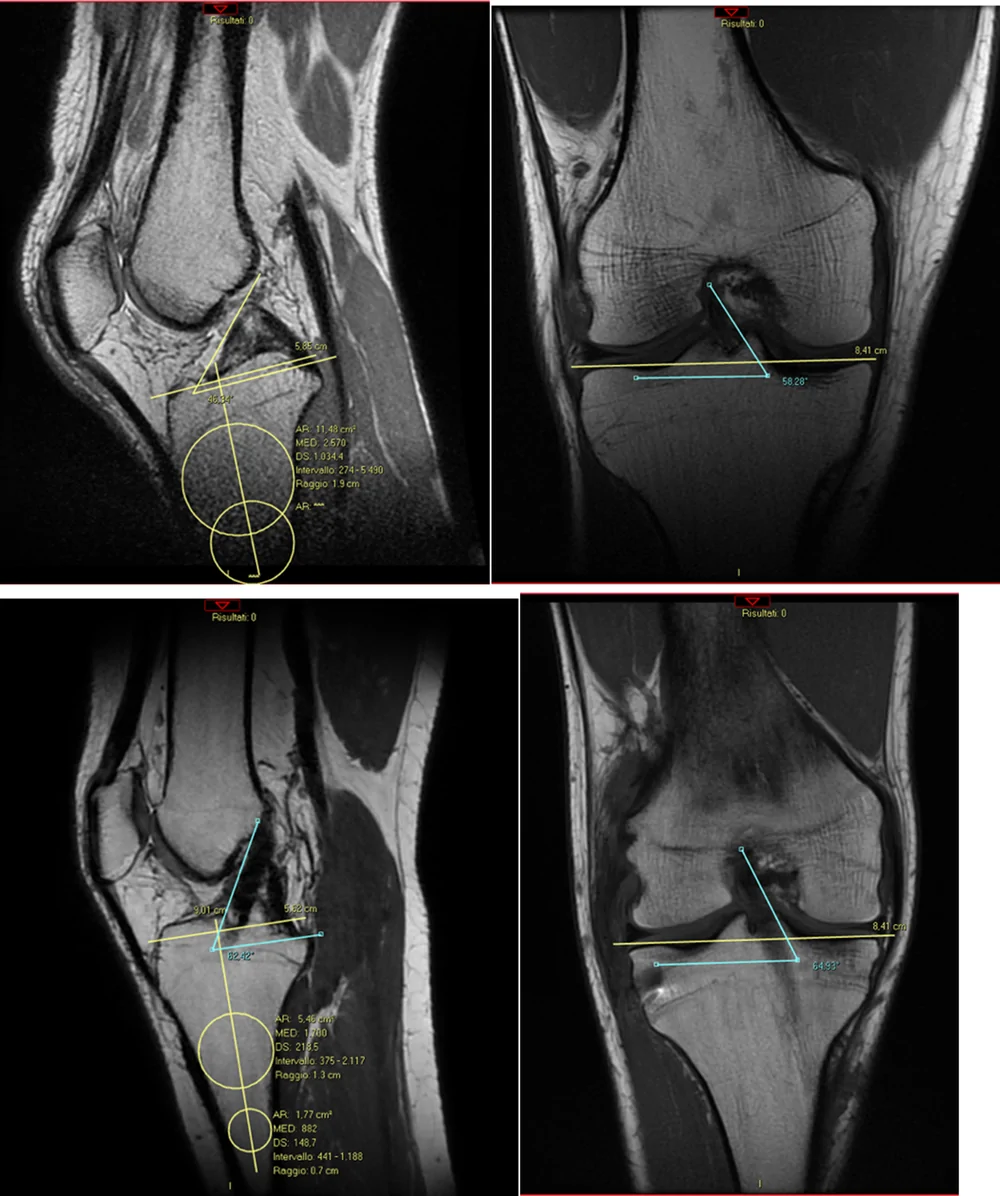
MRI assessment of graft orientation. (a) Sagittal view of the native ACL demonstrating the sagittal inclination angle (SIA) of 46.3°. (b) Coronal view of the native ACL demonstrating the coronal inclination angle (CIA) of 58.3°. (c) Postoperative sagittal MRI following OTT plus LET ACL reconstruction demonstrating the graft SIA of 62.4°. (d) Postoperative coronal MRI demonstrating the graft CIA of 64.9°. ACL, anterior cruciate ligament; LET, lateral extra‐articular tenodesis; MRI, magnetic resonance imaging; OTT, over‐the‐top.

Its durability reflects principles embedded well before they gained widespread acceptance. From its inception, OTT reconstruction combined hamstring autograft with LET, long before contemporary consensus [24] recommendations and the STABILITY trial [3] highlighted the benefits of LET in young patients receiving a hamstring autograft. In addition, the technique avoids creation of a femoral tunnel, thereby eliminating femoral tunnel malposition—now recognised as the leading technical cause of ACL reconstruction failure and a frequent indication for revision [18, 20, 23, 26]. Although historically excluded from radiographic tunnel comparisons, recent magnetic resonance imaging (MRI)‐based assessments demonstrate that OTT graft orientation falls within established anatomic inclination thresholds [25] and is comparable to tibial‐independent drilling techniques, while outperforming traditional transtibial methods [19]. These findings confirm that, despite the absence of a femoral tunnel, the OTT technique reliably reproduces close‐to‐anatomic graft positioning (Figure 1).

In recent years, there has been renewed international interest in this historically time‐tested technique. The team at Rizzoli Orthopedic Institute has been invited worldwide—including to China and India—to perform live demonstration surgeries and share technical expertise with surgeons seeking reproducible alternatives to tunnel‐based reconstruction.

A distinctive feature of the OTT plus LET technique is its mini‐open lateral approach, which provides direct access to the posterior femoral capsule and OTT position. Unlike conventional single‐bundle ACL reconstruction, this extra‐articular component introduces technical steps that may be unfamiliar to surgeons trained exclusively in all‐arthroscopic tunnel‐based techniques. As such, a learning curve exists, particularly in safe lateral window development and posterior capsular access. The purpose of this review is therefore to present 10 practical tips aimed at overcoming this learning curve, minimising intra‐operative complications and optimising outcomes when performing the OTT with LET ACL reconstruction.

### Tip 1: Choose the right OTT plus LET technique modification

At first glance, this tip may appear simplistic: use the OTT plus LET technique. The underlying principle, however, is thoughtful patient selection combined with technical adaptability. OTT reconstruction is versatile across a broad spectrum of patients, and with consistent technique, reliable outcomes [8, 21] can be achieved regardless of age or level of competition. Modifications based on skeletal maturity may be guided by the BABY‐Knee algorithm described by Grassi et al. [4].

**Figure 2:**
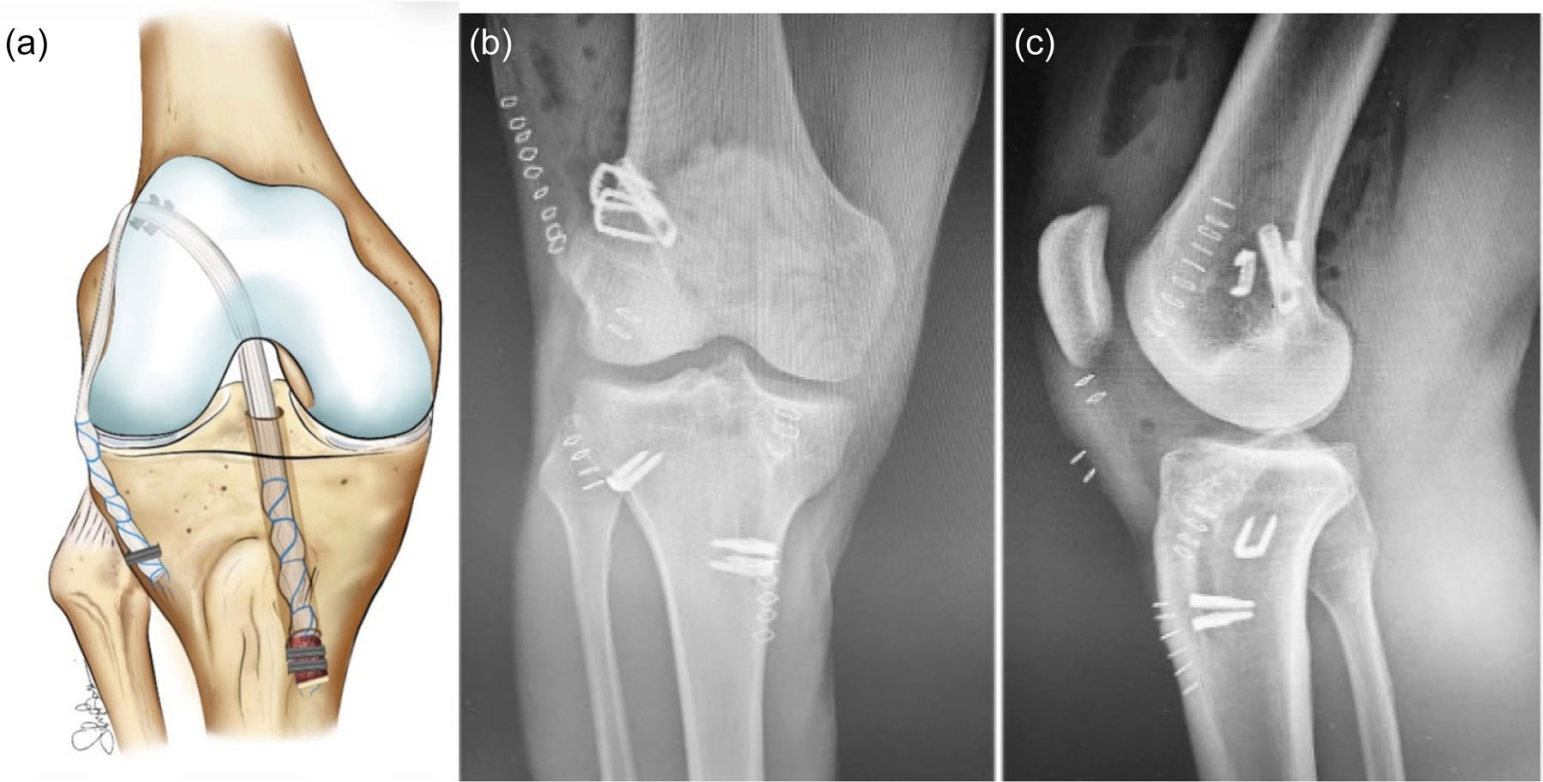
(a) Schematic drawing of revision OTT with LET. (b) Postoperative XR after revision OTT with LET. LET, lateral extra‐articular tenodesis; OTT, over‐the‐top.

In skeletally immature patients, an extra‐physeal WHAT (without hardware and tunnels) variation avoids physeal violation while maintaining rotational control with a lateral plasty [9]. In those nearing skeletal maturity, a supraphyseal technique may be performed under fluoroscopic guidance [5]. For most recreational and elite athletes, the standard OTT technique with hamstring autograft and LET remains appropriate. Return to sport within 6–12 months has been reported in high‐level athletes, with durable long‐term outcomes [1]. The technique is also revision‐friendly [10, 30], as the absence of a femoral tunnel eliminates concerns of tunnel malposition or widening. Revision may be performed using an alternative allograft or autologous rectus femoris tendon using the same technique, with recommended graft length of 22–24 cm (Figure 2).

Mastery across paediatric, primary, elite and revision settings establishes OTT reconstruction as a versatile and reproducible solution for ACL surgery.

### Tip 2: Optimise patient positioning—Supine with figure‐of‐four

Proper patient positioning is fundamental to efficient execution of the OTT plus LET ACL reconstruction. We recommend placing the patient supine on a standard operating table.

**Figure 3:**
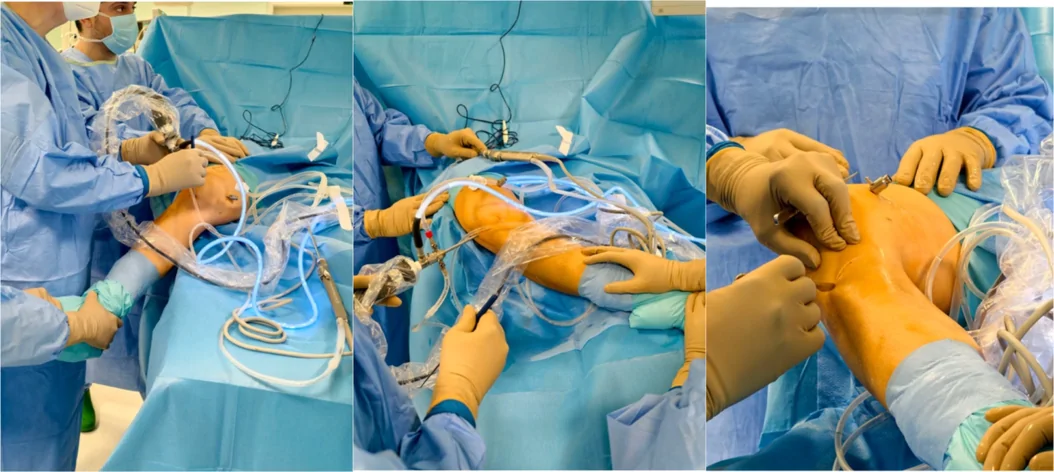
(a) Valgus stress applied using a lateral post positioned at the ipsilateral thigh. (b) Varus stress achieved with the leg placed in the figure‐of‐four position. (c) The supine position with the limb in figure‐of‐four configuration, which provides optimal exposure for hamstring graft harvest.

The supine position first allows optimal management of associated intra‐articular pathology, which commonly accompanies ACL tears. With the use of a side post, the knee can be placed in valgus alignment to open the medial compartment for treatment of medial meniscal tears. Similarly, positioning the knee in a figure‐of‐four configuration opens the lateral compartment, facilitating access to lateral meniscal injuries. This versatility enables comprehensive meniscal management before proceeding to ACL reconstruction (Figure 3).

**Figure 4:**
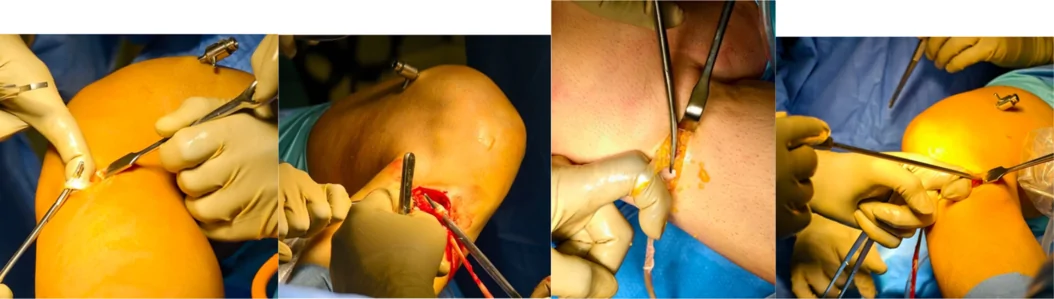
(a) Leg positioned in the figure‐of‐four configuration with identification of the first hamstring tendon. (b) Gracilis harvested with the semitendinosus tendon identified. (c) Accessory vincula incised to prevent premature tendon truncation. (d) Use of an open tendon stripper with the knee maintained in the figure‐of‐four position.

The same positioning then facilitates hamstring graft harvest. With the knee maintained in figure‐of‐four, the pes anserinus is well exposed, allowing clear identification of the gracilis and semitendinosus tendons. This orientation improves visualisation, permits controlled release of accessory vincula and reduces the risk of premature tendon amputation (Figures 3 and 4).

Supine positioning also allows stable and reproducible knee manipulation during later graft passage over the posterior femoral cortex. In contrast, positioning on a break table often requires additional assistance to maintain knee flexion and may introduce unintended motion during critical steps.

Careful attention to positioning streamlines the procedure and enhances reproducibility of the OTT plus LET technique.

### Tip 3: Preserve graft vascularity—Protect the distal hamstring attachment

Preservation of graft vascularity is a defining principle of the OTT plus LET technique and a key biological advantage [27]. Unlike most contemporary autografts, which undergo an early necrotic phase before revascularisation, hamstring grafts with preserved tibial attachment remain biologically viable. MRI studies [6, 13, 14, 22] suggest that vascularises hamstring grafts do not exhibit the same early necrosis, supporting a more favourable healing environment. Preserving the distal attachment is therefore critical.

**Figure 5:**
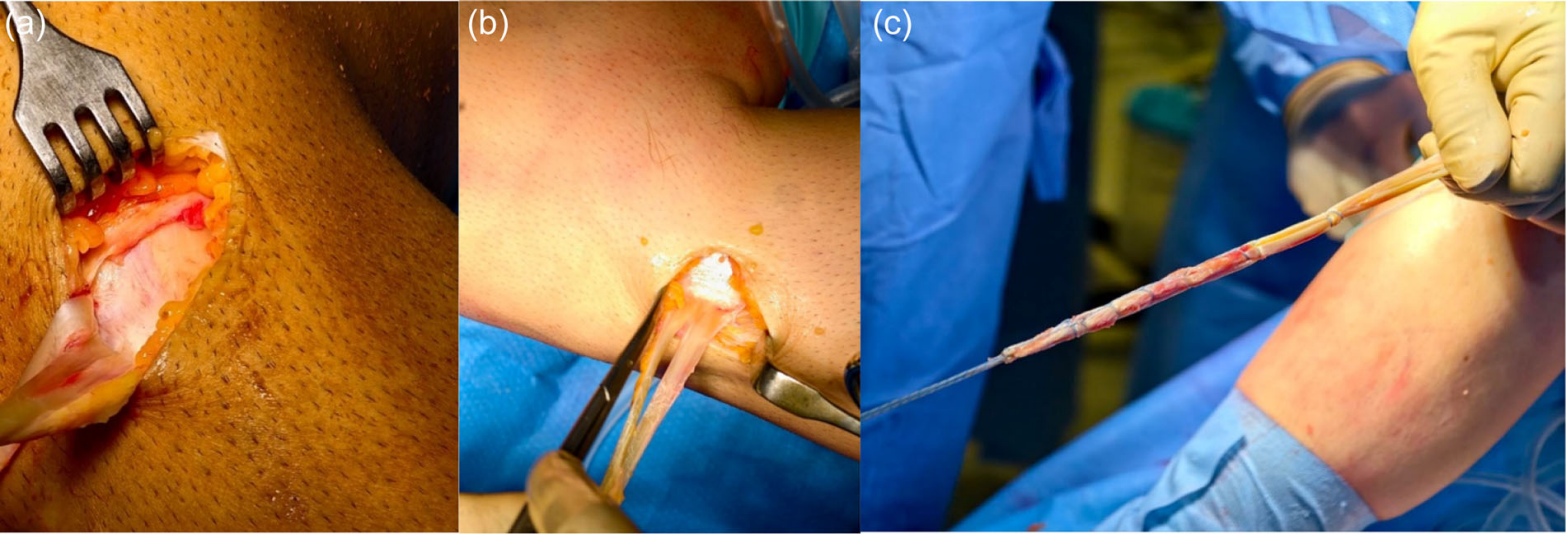
(a) Preservation of graft vascularity with the hamstring tendons maintained at their distal tibial attachment. (b) Distal accessory fibres incised to prevent kinking of the vascular pedicle during graft mobilisation. (c) Two interlocking Krackow stitches placed on the distal one‐third of the graft, with the second stitch capturing both tendons and incorporating the first stitch to prevent differential sliding or bunching during graft passage. Note the interlocking configuration demonstrated using sutures of different colours.

During harvest, the knee is placed in the figure‐of‐four position. The sartorius fascia is incised to identify the semitendinosus and gracilis tendons, which are harvested proximally using an open stripper while maintaining their distal tibial insertion. Distal accessory fibres must be carefully released to prevent tethering or kinking of the vascular pedicle during graft mobilisation (Figure 5).

Graft preparation is equally important. Two interlocking Krackow stitches are placed along the distal one‐third of each tendon, spaced approximately 2 cm apart. It is essential that the second Krackow stitch captures both tendons together, incorporating the first stitch construct, to prevent differential sliding or bunching during graft passage. If the sutures do not secure both tendons as a single unit, the graft may clump as it is shuttled over the posterior femoral cortex, increasing resistance and compromising smooth advancement.

**Figure 6:**
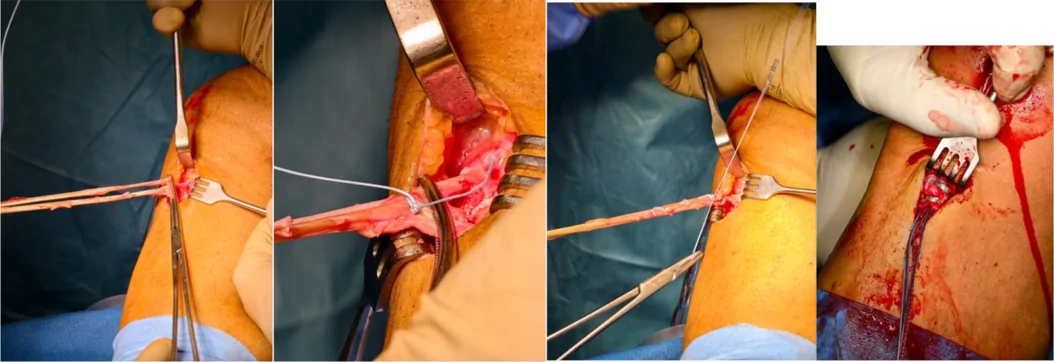
(a) Avulsed tendon positioned alongside the intact tendon using a clamp. (b) Initial suture securing the avulsed tendon to the intact tendon. (c) Completed whipstitch repair of the avulsed tendon to the intact tendon. (d) Use of two 8‐mm staples to reattach both tendons to the medial tibial surface in the rare event that both tendons are avulsed.

Two principal complications may occur during harvest: distal tibial avulsion and proximal truncation. If one tendon is avulsed, it should be sutured side‐to‐side to the intact tendon, with optional staple reinforcement. If both tendons are avulsed, the surgeon may convert to a single‐bundle ACL reconstruction or reattach the graft to the pes anserinus using staples, recognising that vascularity may be altered (Figure 6).

**Figure 7:**
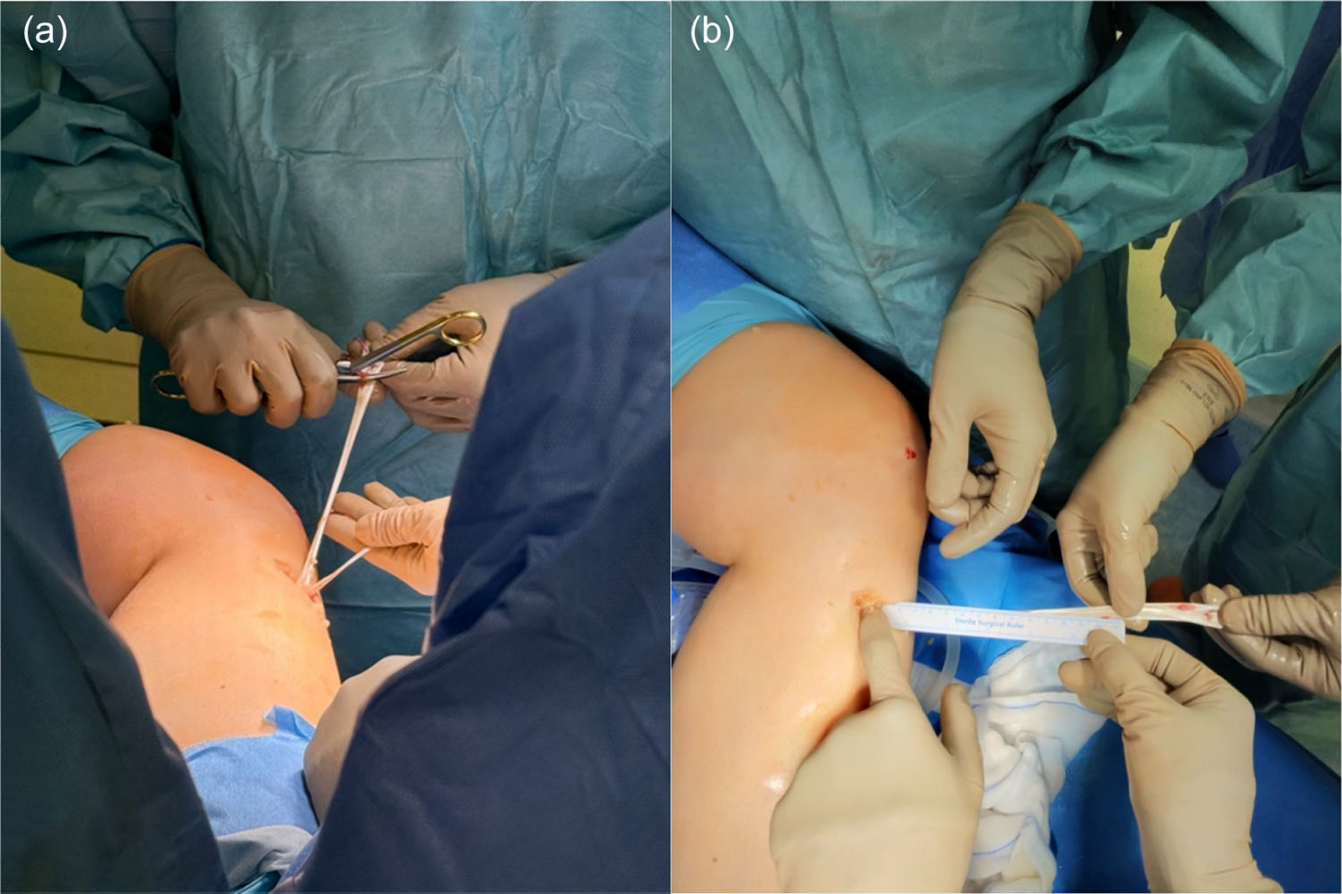
(a) Proximal truncation of one hamstring tendon during harvest. (b) The truncated tendon measuring 15 cm in length.

If proximal truncation occurs in one tendon, the procedure may proceed, accepting a thinner lateral extra‐articular component (Figure 7). If both tendons are truncated and total graft length is less than 20 cm, conversion to a single‐bundle reconstruction or completion with femoral staple fixation and a separate modified Lemaire tenodesis may be considered.

Meticulous harvest and preparation preserve vascularity and maintain one of the fundamental biological advantages of the OTT plus LET reconstruction.

### Tip 4: Adequately clear the OTT femoral passage

Preparation of the OTT femoral pathway is a critical step of the procedure. Inadequate clearance of the medial aspect of the lateral femoral condyle can impede graft passage and compromise final positioning.

The medial wall of the lateral femoral condyle should be thoroughly cleared using a shaver or radiofrequency device under arthroscopic visualisation. All synovium and remnant ACL fibres obstructing the posterior notch must be removed to expose clean cortical bone. The posterior aspect of the condyle should be fully visualised to ensure no soft‐tissue bridge remains that could obstruct smooth graft advancement.

**Figure 8:**
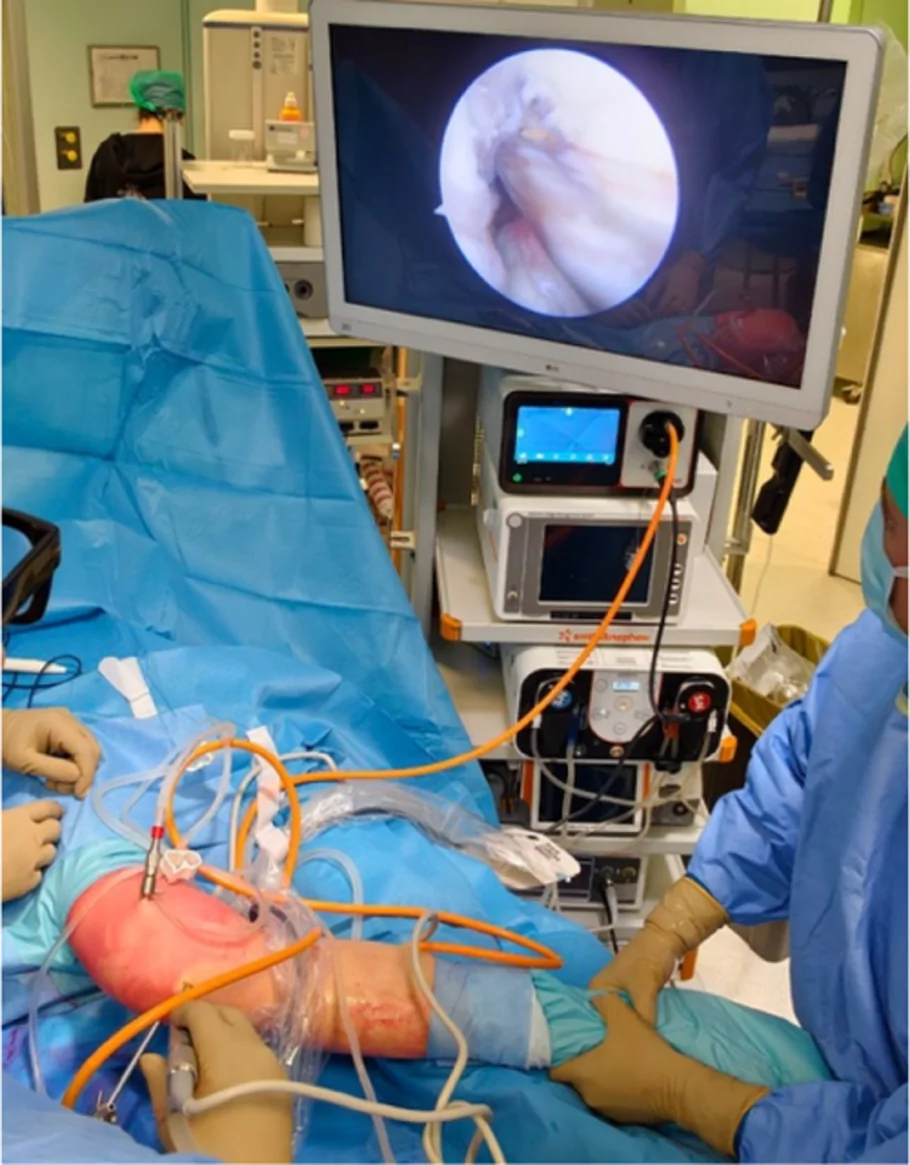
Clearance of the femoral OTT passage performed with the knee positioned in the figure‐of‐four configuration. OTT, over‐the‐top.

This step is best performed with the knee in the figure‐of‐four position (Figure 8), viewing through the anterolateral portal and working through the anteromedial portal. Adequate preparation not only facilitates graft passage but also promotes biological integration, as gentle decortication stimulates bleeding and enhances graft‐to‐bone healing along the posterior cortex.

Meticulous clearance is also essential for anatomic graft orientation. Proper preparation allows the graft to contour naturally around the lateral femoral condyle, reproducing the direct and indirect fibre orientation of the native ACL. This is reflected in postoperative MRI assessments demonstrating appropriate sagittal and coronal inclination angles. In contrast, retained soft tissue may displace the graft inferiorly, decreasing inclination angles and potentially resulting in suboptimal graft biology and impaired graft maturation [12].

Careful preparation of the OTT pathway, therefore, ensures smooth passage, anatomic alignment and optimal graft integration.

### Tip 5: Achieve an anatomic tibial tunnel

**Figure 9:**
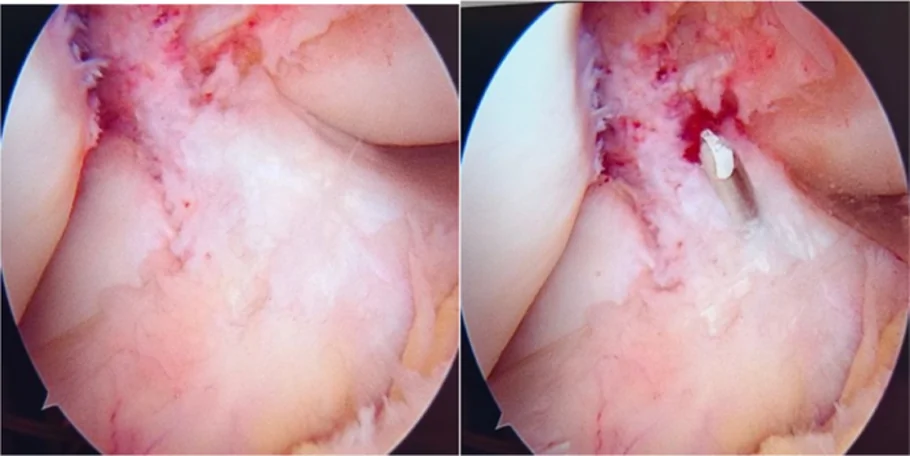
(a) Arthroscopic view demonstrating clear identification of the anterior horn of the lateral meniscus and the ACL stump. (b) Guidewire advanced into the anatomic tibial ACL footprint. ACL, anterior cruciate ligament.

Although the OTT plus LET technique eliminates the need for a femoral tunnel, precise tibial tunnel placement remains essential for restoring graft orientation and preserving biology. The tibial exit should correspond to the native ACL footprint and can be identified using the anterior horn of the lateral meniscus or the remnant ACL stump (Figure 9).

**Figure 10:**
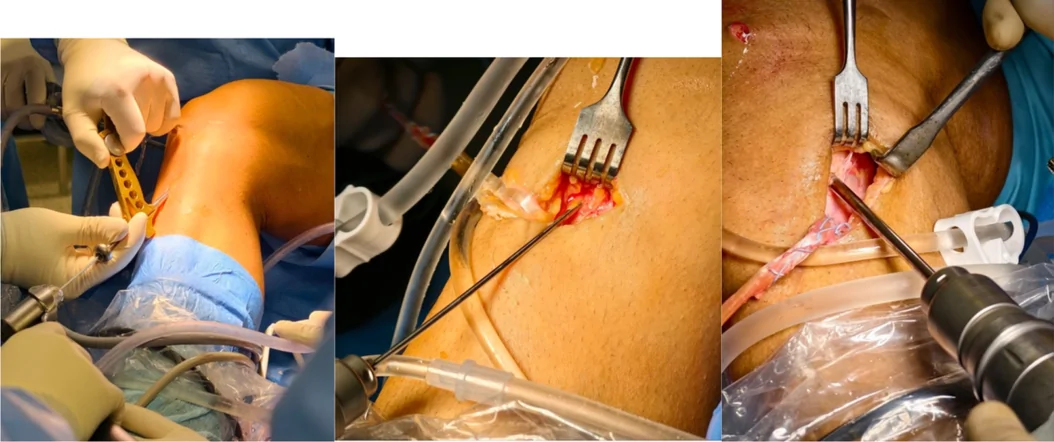
(a) Use of an elbow aimer set to 65°. (b) Guidewire placed on the medial tibial surface approximately 1 cm proximal to the gracilis tendon insertion. (c) The guidewire overdrilled with a 7–8 mm reamer to create the tibial tunnel.

The medial tibial entry point differs from conventional techniques. The tunnel should begin approximately 1 cm proximal to the gracilis tendon attachment along the medial tibial surface (Figure 10). This reduces graft kinking as the vascularises tendons enter the tunnel and preserves vascular continuity. As a result, the tibial tunnel is typically more vertical than in standard single‐bundle reconstruction.

When using an elbow aimer, the guide should be set to 65–70 degrees to achieve an anatomic exit while maintaining a smooth graft trajectory. Minor adjustments can be made using a pilot ream and fine guidewire manipulation before definitive reaming. In most primary cases, a 7‐mm tunnel is sufficient; 8 mm may be required depending on graft size. In revision cases, the tunnel diameter should be individualised and may exceed 9 mm.

**Figure 11:**
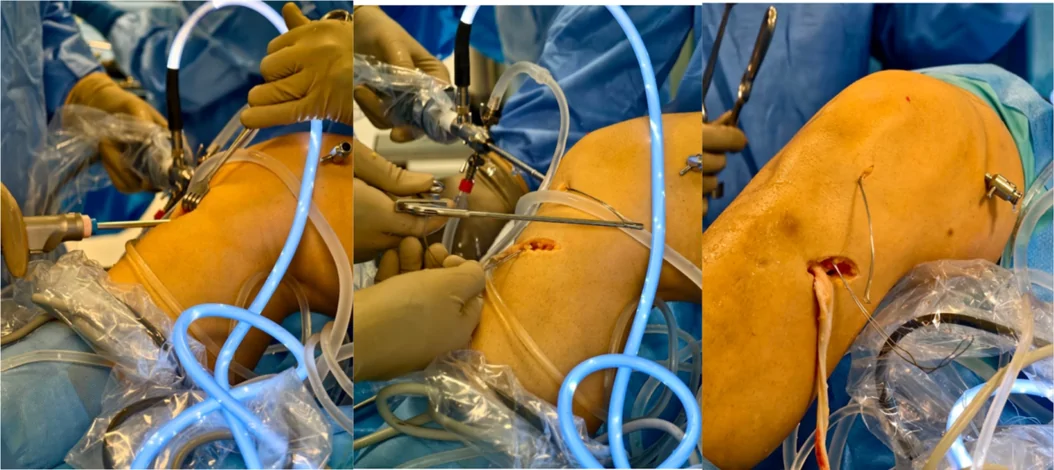
(a) Arthroscopic shaver inserted through the tibial tunnel to remove remnant debris and clear soft tissue from the intra‐articular tunnel aperture. (b) Arthroscopic grasper retrieving the looped passing wire through the anteromedial portal. (c) Final configuration of the looped wire, passed through the tibial tunnel and exiting via the anteromedial portal.

Once the tibial tunnel has been drilled, an arthroscopic shaver should be inserted through the tunnel to remove remnant debris and bone fragments (Figure 11). This step ensures that the intra‐articular aperture of the tibial tunnel is completely free of soft tissue and obstruction. Clearing the tunnel at this stage facilitates smooth graft passage, reduces friction at the tunnel entrance and minimises the risk of graft impingement.

After tunnel preparation, a looped passing wire is introduced through the tunnel and retrieved through the anteromedial portal using a grasper. The looped end is brought out through the portal, while the loose end of the wire emerges from the distal opening of the tibial tunnel. The wire is left in situ to serve as a shuttle for subsequent suture and graft passage later in the procedure.

Meticulous tibial tunnel placement ensures anatomic alignment, preserves graft vascularity and enhances reproducibility of the OTT plus LET reconstruction.

### Tip 6: Develop the window safely

The lateral approach is the foundation for safe graft passage and femoral fixation. Proper exposure and controlled development of the OTT window are essential.

The lateral incision begins at the level of the lateral epicondyle and extends approximately 5 cm proximally along the axis of the femur. The skin incision is placed approximately 1 cm posterior to the palpable posterior border of the iliotibial band (ITB) with the knee flexed at 50 degrees. After clearing the subcutaneous tissue, the ITB is clearly identified, and a longitudinal incision is made in line with the skin incision, again approximately 1 cm anterior to its posterior border.

Direction of tissue retraction is paramount at this stage. Although it may seem intuitive for the assistant to retract the posterior leaflet of the ITB directly posteriorly, doing so obscures visualisation of the Kaplan fibres. Instead, the assistant should retract the posterior ITB leaflet laterally. This manoeuvre exposes the Kaplan fibres clearly, allowing them to be visualised as the floor of the operative field and facilitating controlled release.

**Figure 12:**
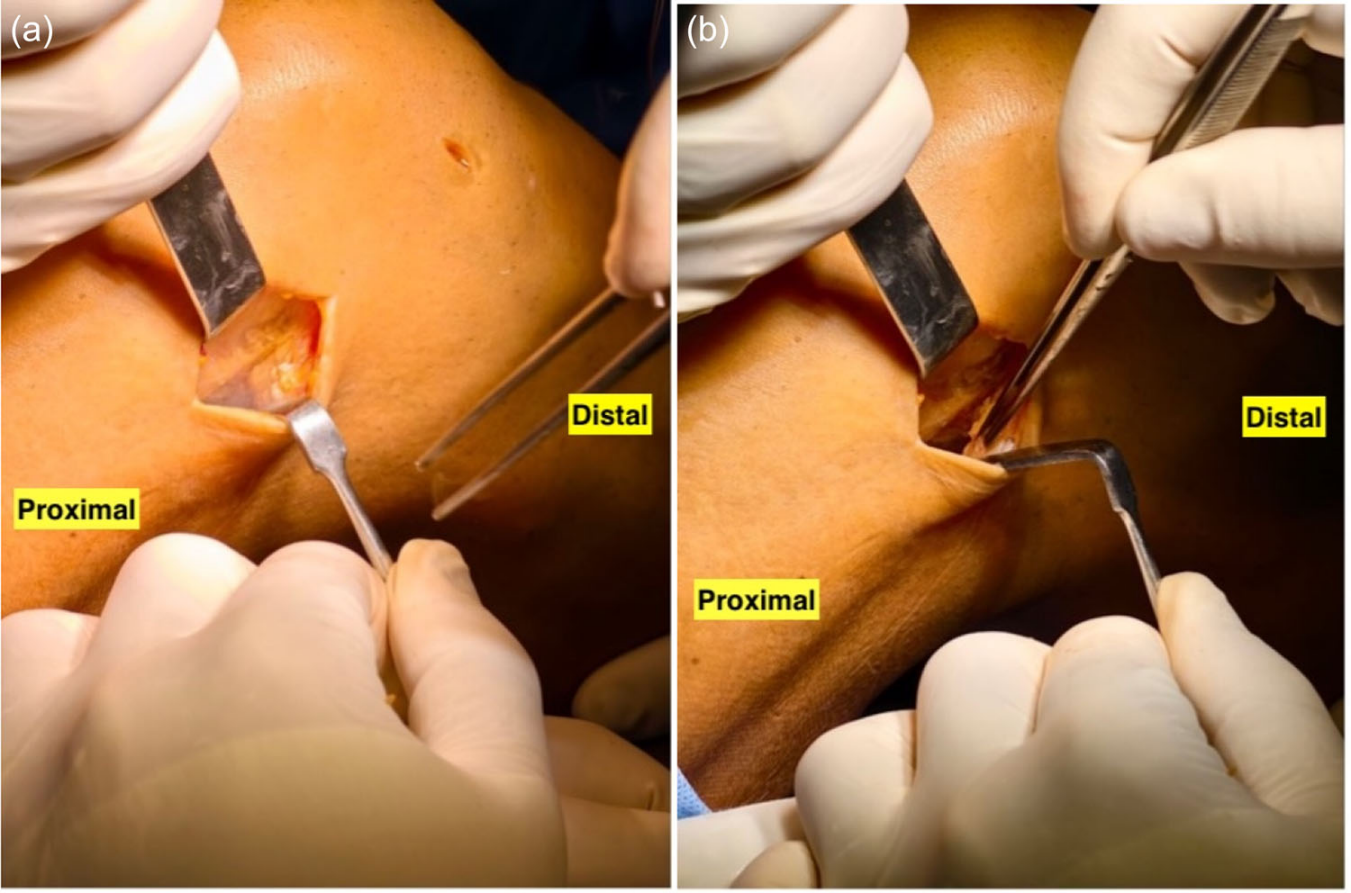
(a) Kaplan fibres visualised along the deep surface of the iliotibial band (ITB) when the posterior leaflet of the ITB is retracted laterally. (b) Kaplan fibres incised with diathermy, revealing the over‐the‐top window; forceps demonstrating the released fibres attached to the ITB.

Attention is then directed to the Kaplan fibres, which arise from the deep surface of the ITB and insert onto the distal femur in the supracondylar region. These fibres must be carefully released to create a safe plane toward the posterior capsule (Figure 12). The release should be performed using diathermy as close to the ITB as possible to minimise the risk of injury to the lateral superior geniculate artery, which courses adjacent to the femoral cortex. The fibres are typically divided at approximately a 45‐degree angle, allowing a controlled opening of the interval.

Once divided, a natural plane develops toward the posterior femoral capsule. A finger is inserted in a hooked manner to gently develop this plane. With careful palpation, the posterior aspect of the lateral femoral condyle and the entrance to the intercondylar notch can be identified, confirming correct positioning and establishing a safe corridor for clamp passage.

Meticulous development of this window protects vascular structures, respects the anterolateral anatomy and creates the anatomical pathway required for controlled OTT graft passage.

### Tip 7: Safe passage of Kelly Clamp using tactile feedback

Clamp passage through the posterior capsule into the OTT position must be deliberate and controlled, with protection of the posterior cruciate ligament (PCL) and posterior neurovascular structures as the primary concern.

The knee should be maintained at approximately 90° of flexion throughout this step. Studies [2, 11] evaluating posterior safety during total knee arthroplasty and meniscus repair have shown that flexion allows the neurovascular bundle to fall posteriorly, increasing the safety margin during capsular perforation.

**Figure 13:**
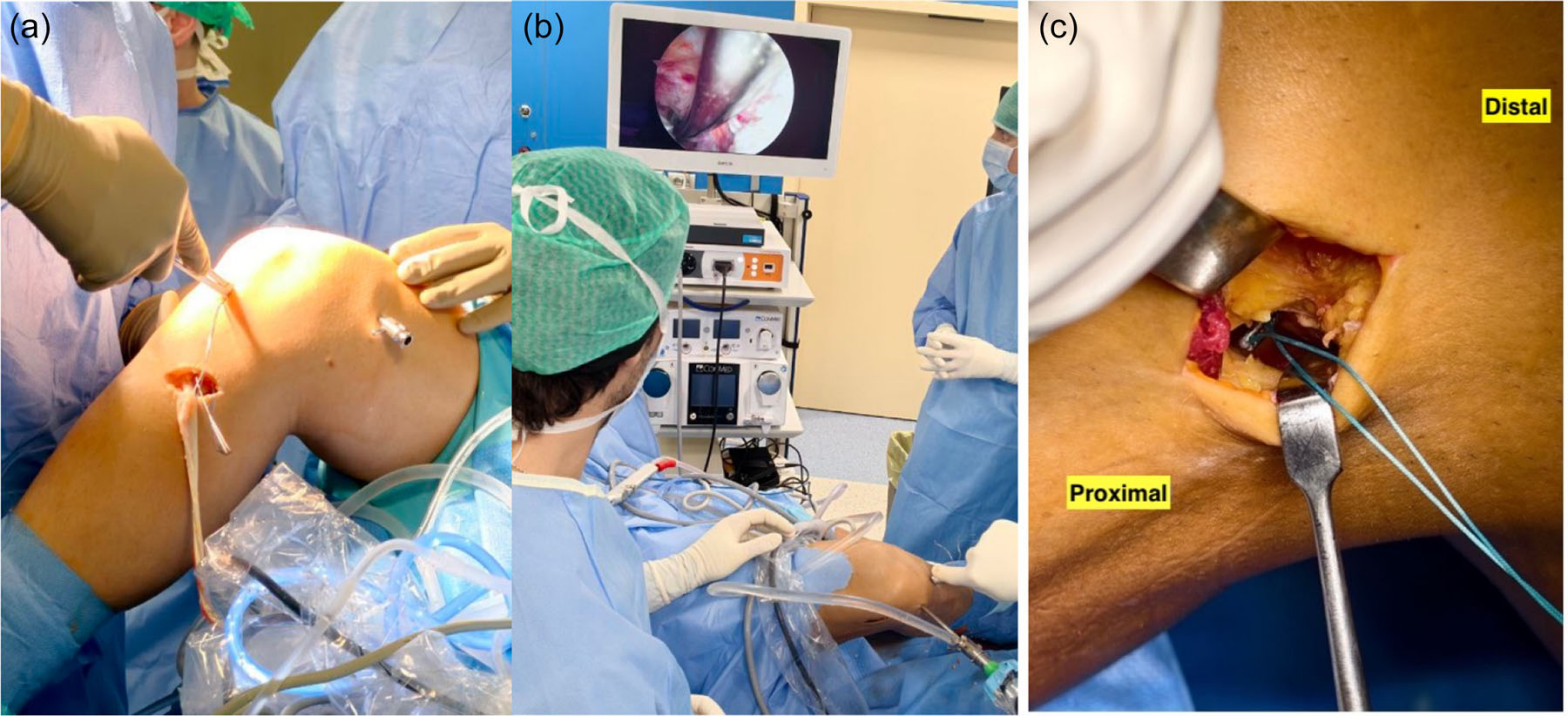
(a) Kelly clamp passage guided by tactile feedback, with the tip maintained in constant contact along the medial surface of the lateral femoral condyle and confirmed by fingertip palpation through the lateral incision. (b) Arthroscopic assistance used to visualise the intercondylar notch and avoid the posterior cruciate ligament (PCL) during clamp advancement. (c) Controlled perforation of the posterior capsule with shuttle sutures prepared for passage.

The Kelly clamp is introduced through the anteromedial portal and advanced toward the posterior aspect of the lateral femoral condyle (Figure 13). Continuous bony contact is essential: the curved tip should slide along the medial surface of the lateral femoral condyle. The surgeon's finger in the lateral incision must feel the clamp advancing onto the fingertip, confirming a safe lateral trajectory. The instrument must not stray medially toward the PCL.

If resistance or uncertainty arises, advancement should stop immediately. A dry arthroscope introduced through the anterolateral portal can confirm correct clamp position before controlled capsular perforation.

Once the clamp exits through the lateral incision, the looped shuttle suture is passed through its jaws and the clamp is withdrawn through the anteromedial portal, retrieving the looped suture.

Safe clamp passage relies on three principles: knee flexion, continuous bony contact with tactile confirmation and readiness to use arthroscopic visualisation when in doubt.

### Tip 8: Controlled graft passage

At this stage, the OTT window and shuttle system are in place, but the pathway is not fully established until the shuttle suture is cycled. The looped shuttle suture is retrieved distally through the tibial tunnel using the previously placed passing wire. Before graft passage, it must be cycled in a controlled back‐and‐forth motion to dilate the posterior capsule and confirm unobstructed passage.

**Figure 14:**
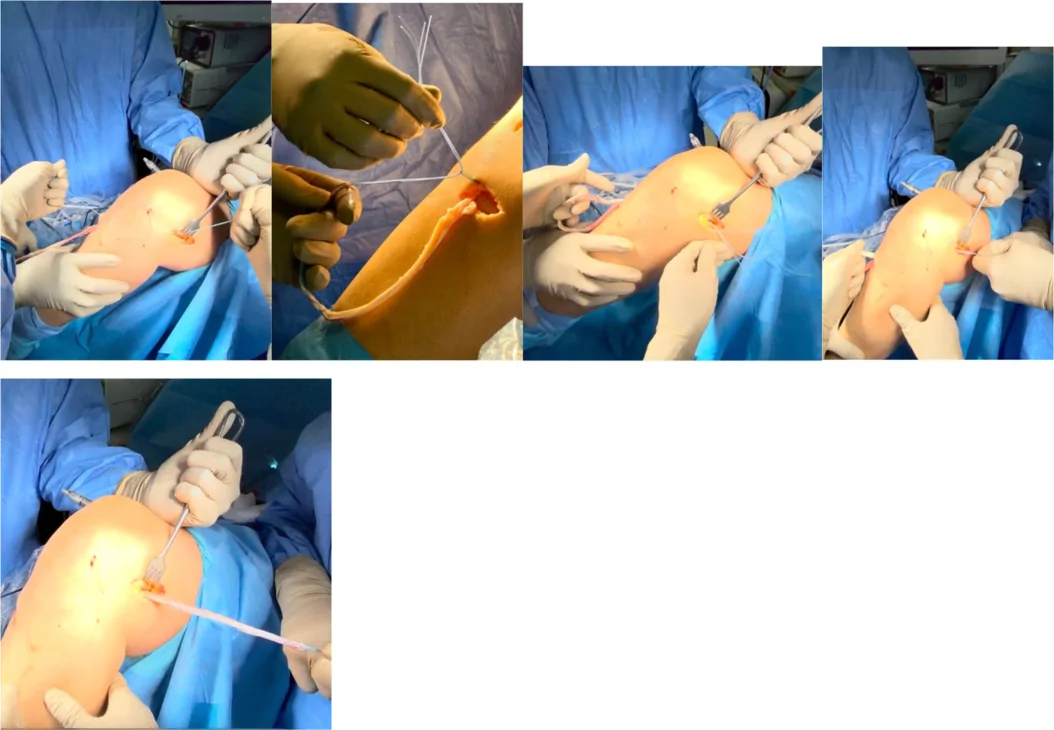
(a) Shuttle suture traversing the tibial tunnel and lateral incision is cycled in a controlled back‐and‐forth motion to dilate the posterior capsular opening. (b) Krackow sutures of the graft secured to the looped shuttle suture. (c) Krackow sutures delivered through the lateral incision. (d) Controlled advancement of the graft through the tibial tunnel and over‐the‐top pathway. (e) Graft successfully positioned across the tibial tunnel and over‐the‐top femoral surface.

A dry arthroscope may be introduced through the anterolateral portal. Graft passage occurs in two phases. First, only the Krackow sutures are shuttled across to ensure smooth traversal of the tibial tunnel and posterior femoral cortex (Figure 14). Second, with the sutures exiting laterally, controlled distal tension is applied while the sutures are pulled to progressively draw the graft across the joint and over the lateral femoral condyle.

Traction should be smooth and never forceful. Resistance usually indicates graft bunching; the graft should be retrieved, the Krackow sutures revised if necessary, and passage repeated. Throughout advancement, the distal hamstring attachment must remain untwisted to preserve vascularity.

Controlled graft passage—guided by visualisation and restraint—prevents mechanical injury and preserves the biological advantages of the OTT plus LET reconstruction.

### Tip 9: Insert femoral staples safely

Femoral fixation in the OTT technique depends on precise staple placement. Two 8‐mm titanium toothed staples are recommended. The toothed design penetrates tendon and cortical bone, creating compression that promotes bleeding and graft‐to‐bone integration along the posterior femoral surface. Before definitive femoral fixation, the tourniquet should be released and the lateral wound carefully inspected for bleeding, particularly from the lateral superior geniculate artery, which may have been at risk during Kaplan fibre release. Haemostasis must be achieved before staple insertion to avoid postoperative haematoma and ensure clear visualisation during fixation.

**Figure 15:**
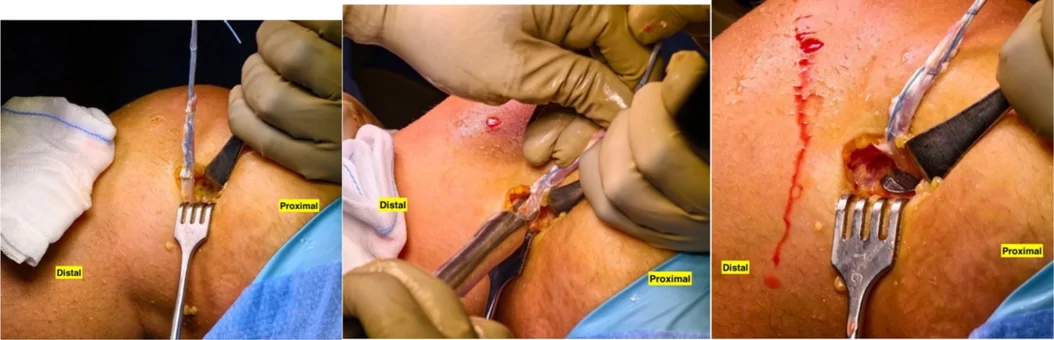
(a) Graft reflected anteriorly to expose the underlying femoral cortex, with periosteum cleared using diathermy to prepare the fixation surface. (b) Graft repositioned vertically, perpendicular to the femoral shaft axis, over the exposed cortical bone prior to insertion of the first 8‐mm staple. (c) Final configuration demonstrating placement of two 8‐mm staples securing the graft in the over‐the‐top position.

The graft is then held vertically, perpendicular to the femoral shaft, to define its borders (Figure 15). The periosteum is cleared with diathermy to expose clean cortical bone before fixation (Supplementary Video S1). Proper tensioning requires coordinated assistance: posterior drawer is applied with the tibia in external rotation while vertical traction is maintained on the graft to ensure it lies flush against the posterior cortex. As a practical guide, the appropriate tension is approximately that required to just lift the foot off the operating table when the knee is flexed to 90° and the graft is tensioned vertically.

The first staple is placed at the junction of the middle and posterior thirds of the lateral femoral cortex and directed anteriorly and distally to avoid posterior cortical blowout and protect the neurovascular structures. The second staple is typically inserted inferior to the first staple, maintaining the same anterior‐distal orientation. This vertical stacking configuration provides secure compression while preserving a safe posterior margin.

After placement of the first staple, its position should be palpated with reference to the posterior border of the femur to confirm that it is not excessively posterior. If the first staple is found to be positioned more posteriorly than intended, the second staple should be inserted superior to the first rather than inferiorly. This adjustment prevents unintended posterior convergence and reduces the risk of posterior cortical breach.

**Figure 16:**
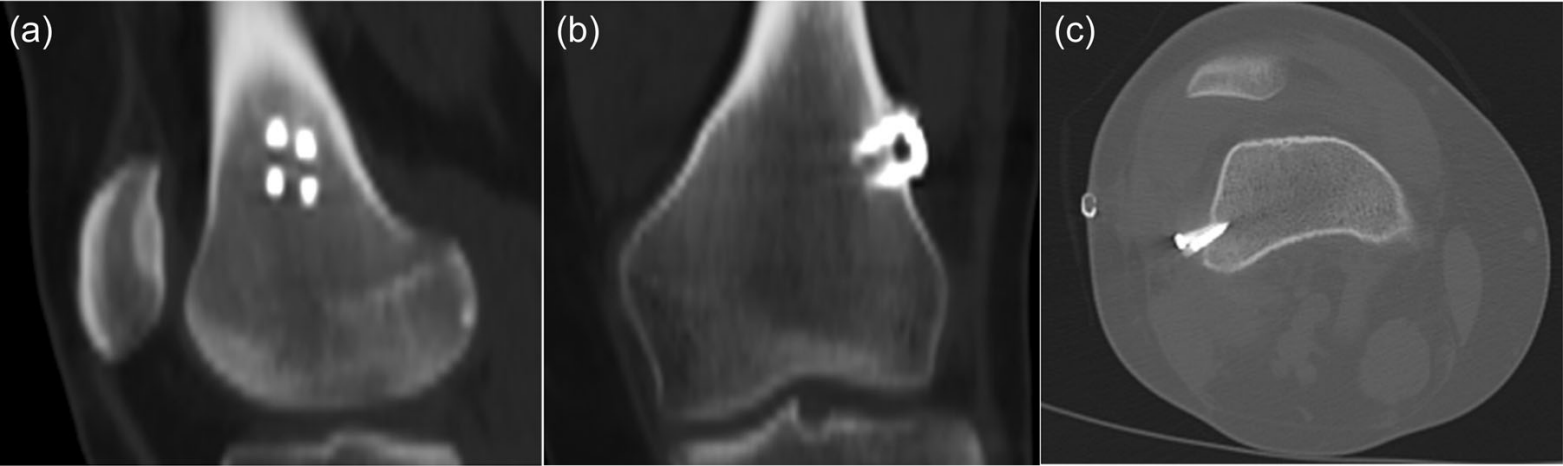
Computed tomography (CT) images following over‐the‐top plus LET ACL reconstruction. (a) Sagittal view demonstrating ideal femoral staple placement along the posterior aspect of the distal femur. (b) Coronal view showing staple position relative to the distal femoral condylar flare, with distal angulation. (c) Axial view illustrating posterior staple placement with anterior‐directed angulation.

Both staples must maintain the anterior‐distal trajectory to ensure stable fixation while protecting posterior structures (Figure 16).

Excessive force may cause graft truncation. If the intra‐articular portion remains secure, fixation may proceed with the posterior drawer maintained, and the LET completed using another technique such as a modified Lemaire's. If the graft dislodges but sufficient length remains, it may be whip‐stitched and re‐fixed. If the graft length is inadequate, conversion to single‐bundle reconstruction with suspensory fixation and LET, or use of an alternative graft (quadriceps, rectus femoris, or bone‐patellar‐tendon‐bone), should be considered.

Precise staple orientation, controlled tensioning and avoidance of force are critical to stable fixation while preserving the biological advantages of the OTT plus LET reconstruction.

### Tip 10: Complete the LET

The final step of the technique is the completion of the LET, which reinforces rotational control and protects the intra‐articular graft.

A diagonal incision is made just distal to Gerdy's tubercle to avoid hardware prominence. Positioning fixation below the tubercle allows the bony prominence to shield the implant and reduce irritation.

**Figure 17:**
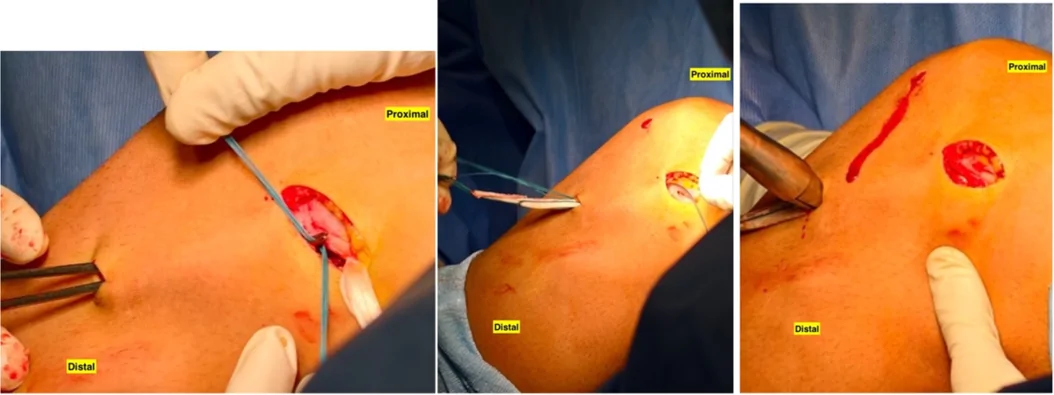
(a) Artery clamp passed deep to the iliotibial band (ITB) from distal to proximal. (b) Graft of sufficient length positioned beyond Gerdy's tubercle prior to fixation. (c) Insertion of a 6‐mm toothed staple to complete the lateral extra‐articular tenodesis.

**Figure 18:**
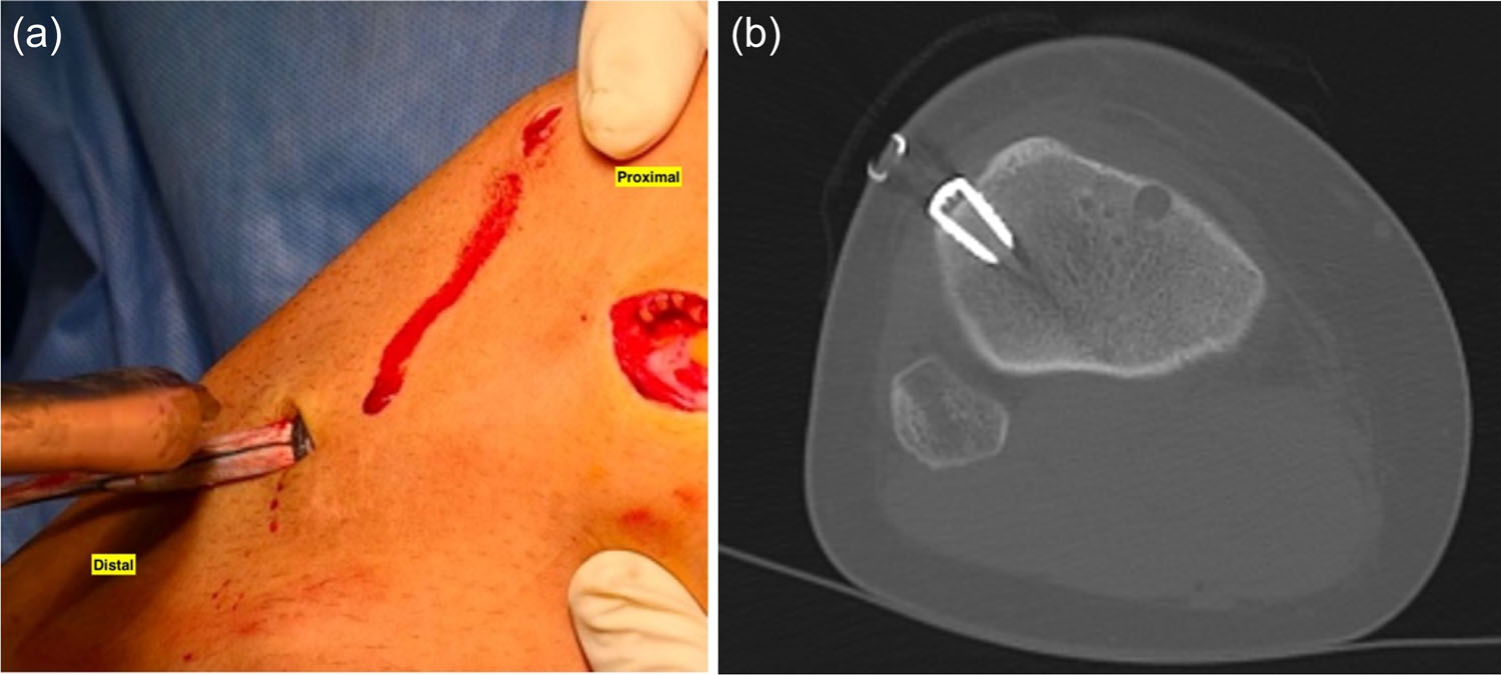
(a) Final position of the tibial bone staple following fixation. (b) Axial CT view demonstrating ideal placement of the tibial staple seated against the lateral tibial cortex.

The incision is carried down to bone, and a clamp is passed from distal to proximal beneath the ITB and retrieved through the proximal lateral incision, ensuring the graft lies in the correct extra‐articular plane. After confirming proper positioning, the Krackow sutures are tensioned distally while the assistant returns the tibia to neutral rotation and maintains the posterior drawer. A 6‐mm titanium toothed staple is then inserted for tibial fixation (Figures 17 and 18).

**Figure 19:**
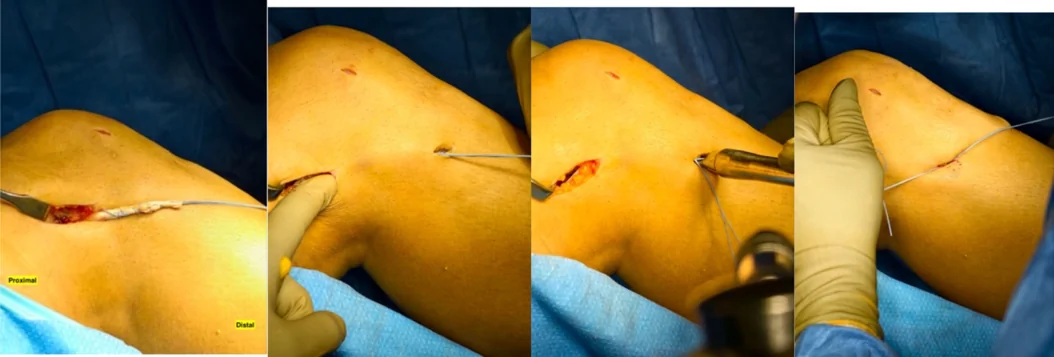
(a) Estimated graft length appears marginal for completion of the lateral extra‐articular tenodesis. (b) Graft passed beneath the iliotibial band (ITB) but not reaching Gerdy's tubercle. (c) Two of the four Krackow sutures secured beneath the staple. (d) Remaining sutures tied over the staple in a staple–suture construct to complete fixation.

If graft length is marginal, part of the Krackow sutures may be secured beneath the staple and the remaining limbs tied over it (Figure 19). If a graft–tibia gap greater than 2 cm is identified, a separate LET procedure may be considered, although this is rarely required.

Completion of the extra‐articular component restores rotational stability, protects the intra‐articular graft during early healing, and reinforces the durability of the OTT plus LET technique.

## CONCLUSION

OTT plus LET ACL reconstruction has remained in continuous use for more than three decades, with durable clinical outcomes across paediatric, primary, elite and revision populations. Its longevity is not incidental, but grounded in reproducible surgical principles that prioritise graft biology, anatomic alignment, and protection of posterior structures while eliminating femoral tunnel–related technical errors.

The 10 technical recommendations presented in this review reflect cumulative institutional experience and are designed to prevent and manage intra‐operative complications. Particular emphasis is placed on preservation of hamstring vascularity, precise tibial tunnel placement, meticulous preparation of the OTT femoral pathway, controlled graft passage, accurate staple orientation and standardised completion of the lateral extra‐articular component.

When performed with attention to these details, the OTT plus LET technique provides reliable fixation, reproducible close‐to‐anatomic graft orientation and a biologically favourable construct. Adherence to these principles allows surgeons to safely expand the application of this technique across a broad patient spectrum while minimising technical failure.

## AUTHOR CONTRIBUTIONS

Giulio Maria Marcheggiani Muccioli initiated the manuscript. Mok Ying Ren drafted the manuscript. Alberto Grassi, Giulio Maria Marcheggiani Muccioli and Stefano Zaffagnini were the lead surgeons responsible for the surgical procedures and articulating the tips. All authors critically reviewed the manuscript and approved the final version.

## FUNDING INFORMATION

The authors have no funding to report.

## CONFLICTS OF INTEREST STATEMENT

Alberto Grassi, MD: Smith & Nephew: Not paid consultant. Stefano Zaffagnini, MD, Professor: DePuy, a Johnson & Johnson Company: Paid presenter or speaker; paid consultant, European Society of Sports Traumatology, Knee Surgery and Arthroscopy (ESSKA): Board or committee member, International Society of Arthroscopy, Knee Surgery, and Orthopaedic Sports Medicine (ISAKOS): Board or committee member, Journal of Experimental Orthopaedics (JEO): Editorial or governing board and Smith & Nephew: Paid presenter or speaker; paid consultant.

## ETHICS STATEMENT

Please include the name of the Institutional Review Board (IRB) and the approval number. If not applicable, please state so.

## Supporting information

Supporting File 1
